# Development of a Melting Curve-Based Allele-Specific PCR of Apolipoprotein E (APOE) Genotyping Method for Genomic DNA, Guthrie Blood Spot, and Whole Blood

**DOI:** 10.1371/journal.pone.0153593

**Published:** 2016-04-14

**Authors:** Chia-Hsiang Chen

**Affiliations:** 1 Department of Psychiatry, Chang Gung Memorial Hospital-Linkou, Taoyuan, Taiwan; 2 Department and Graduate Institute of Biomedical Sciences, Chang Gung University, Taoyuan, Taiwan; University of Navarra, SPAIN

## Abstract

Genetic polymorphisms of apolipoprotein E (APOE) are associated with various health conditions and diseases, such as Alzheimer’s disease, cardiovascular diseases, type 2 diabetes, etc. Hence, genotyping of APOE has broad applications in biomedical research and clinical settings, particularly in the era of precision medicine. The study aimed to develop a convenient and accurate method with flexible throughput to genotype the APOE polymorphisms. A melting curve-based allele-specific PCR method was developed to genotype two single nucleotide polymorphisms (SNPs) of APOE, i.e. rs429358 at codon 112 and rs7412 at codon 158. These two SNPs determine the genotype of APOE2, E3, and E4. PCR-based Sanger sequencing was used as the reference method for APOE genotyping. A 100% concordance rate was obtained in 300 subjects between the melting curve-based allele-specific PCR method and the Sanger sequencing method. This method was applied to a genetic association analysis of APOE and schizophrenia consisting of 711 patients with schizophrenia and 665 control subjects from Taiwan. However, no significant differences in the allele and genotype frequencies were detected between these two groups. Further experiments showed that DNA dissolved from blood collected on Guthrie filter paper and total blood cell lysate without DNA extraction can be used in the melting curve-based allele-specific PCR method. Thus, we suggest that this is a fast, accurate and robust APOE genotyping method with a flexible throughput and suitable for DNA template from different preparations. This convenient method shall meet the different needs of various research and clinical laboratories.

## Introduction

APOE gene encodes the apolipoprotein E, which is involved in the transportation of cholesterol and lipids in blood circulation and central nervous system. Apolipoprotein E also mediates lipoprotein internalization and degradation via receptor-mediated endocytosis pathway and plays a crucial role in lipid homeostasis. There are three major isoforms of APOE gene, E2, E3, and E4, which are determined by the genetic polymorphisms at codon 112 (rs429358) and codon 158 (rs7412). APOE2 has a cysteine at both codon 112 (TGC) and codon158 (TGC), APOE3 comprises cysteine at codon 112 (TGC) and arginine at codon 158 (CGC) while APOE4 contains arginine at both codon 112 (CGC) and codon 158 (CGC). There are functional differences in their binding ability to lipids and receptors among different isoforms of apolipoprotein E [1–3].

These genetic polymorphisms of APOE gene are associated with cardiovascular diseases and neurodegenerative diseases [3, 4]. Most notably, APOE4 is associated with elevated risk of Alzheimer’s disease, which has repeatedly been observed across different populations worldwide [5–7]. With the expanding knowledge of the function of apolipoprotein, APOE has also been implicated in the neurobiology of psychiatric disorders [8, 9]. Genetic association studies of APOE variants have been conducted in various psychiatric illnesses, such as major depressive disorder [10, 11], schizophrenia [12–14], bipolar disorder [15–17], autism [18–20], attention deficit hyperactivity disorder [21], panic disorder [22], and post-traumatic stress disorder [23, 24]. In addition to the association with disease risk, genetic polymorphisms of APOE were also associated with efficacy and adverse effects of certain drugs, outcome and prognosis of some diseases [3, 25, 26]. Hence, genotyping of APOE has broad applications in biomedical research, particularly in the era of precision medicine [3].

There are several APOE genotyping methods available at present, such as PCR-based restriction fragment length polymorphism (RFLP) analysis, fluorescent resonance energy transfer (FRET)-based melting curve analysis, TagMan genotyping method, high-resolution melting (HRM) analysis, and PCR direct sequencing [27–29]. These methods have different requirements, advantages, and limitations. Hence, there is a need for a convenient method that can be quickly adopted in various laboratories. Here, we report the development of a closed tube PCR-based APOE genotyping method using fluorescence melting curve analysis with Tm-shift primers. We demonstrated that this method is fast, accurate, and robust and has a flexible throughput that can be used efficiently in different research and clinical laboratories.

## Materials and Methods

### Subjects

DNA samples used in this study were taken from our previous collection for series of the molecular genetic study of schizophrenia. All the subjects were Han Chinese from Taiwan. In brief, genomic DNA was prepared from peripheral blood cells using Gentra Puregene Blood kit according to the manufacturer’s instructions (Qiagen, Hilden, Germany). This study comprised DNA samples of 711 patients with schizophrenia and 665 control subjects collected from for our serial genetic study of schizophrenia. The study was approved by the Medical Research Committee of National Health Research Institutes of Taiwan, and written informed consent was obtained from each participant and their guardians after the whole procedures were fully explained.

### Melting curve-based allele-specific PCR for APOE genotyping

We developed a melting curve-based allele-specific PCR method to genotype APOE polymorphisms. The method mainly followed the principle of PCR Tm-shift SNP genotyping method described by Wang and colleagues with modification [30]. In brief, for the genotyping of the codon 112 (rs429358), a 20 μl PCR mixture containing genomic DNA 100 ng, 0.5 μM of each of the APOE-112-F-T, APOE-112-F-C, and APOE-112-R primers, and 1X SYBR Green PCR Master Mix (Life Technologies, CA, USA) was prepared. For the genotyping of the codon 158 (rs7412), a 20 microliter PCR mixture containing genomic DNA 100 ng, 0.5 micromolar of each of the APOE-158-F-C, APOE-158-F-T, and APOE-158-R primers, and 1X SYBR Green PCR Master Mix (Life Technologies, CA, USA) was prepared. PCR was performed with the initial denaturation at 95°C for 5 minutes and then followed by 35 cycles of denaturation at 95°C 1 minute, annealing and extension at 70°C 30 seconds. After PCR, melting curve analysis was performed from 60°C to 95°C with the ramping of 0.3% per minute using the continuous monitor mode. PCR and melting curve analysis were implemented using the Applied Biosystems StepOnePlus Real-Time PCR Systems following the manufacturer’s protocol (Applied Biosystems, Forster City, California, USA). The sequences of the PCR primers and the size of each PCR product are listed in Table 1.

**Table 1:** Sequences of primers, annealing temperatures and the sizes of PCR product in this study.

Prime name	Sequences	Size (bp)
APOE-112-F-C	5’-gcgggcagggcggcGCGCGGACATGGAGGACGTGC-3’	62
APOE-112-F-T	5’-gcgggcGCGCGGACATGGAGGACGTGT-3’	54
APOE -112-R	5’-CGCCGCGGTACTGCACCAGG-3’	
APOE-158-F-C	5’-gcgggcagggcggcGCCGATGACCTGCAGAAGC-3’	65
APOE-158-F-T	5’-gcgggcGCCGATGACCTGCAGAAGT-3’	57
APOE-158-R	5’-CTCGCGGGCCCCGGCCTGGTA -3’	
APOE-Seq-F	5’- GACCATGAAGGAGTTGAAGGCCTA C-3’	307

The lower case indicates GC tails tagged to the forward primers.

### Sanger sequencing for APOE genotyping

PCR-based Sanger sequencing was used as the reference method to validate the authenticity of the melting curve-based allele-specific PCR method of APOE genotyping. APOE-Seq-F and APOE-158-R primers were used to generate PCR amplicon covering the codon 112 (rs429358) and 158 (rs7412). A 20 microliter PCR mixture containing 100 ng genomic DNA, 0.5 micromolar of each of the APOE-Seq-F and APOE-158-R primers, 0.0125U of KOD FX DNA polymerase (Toyobo Co., Ltd., Osaka, Japan), 0.1 mM dNTP, and 1X KOD FX buffer was prepared. After initial denaturation at 95°C for 5 minutes, 30 cycles of PCR were performed at 95°C 30 seconds, 63°C 30 seconds, and 72°C 30 seconds. Aliquots of PCR product were processed using a PCR Pre-Sequencing Kit (USB Corp. Cleveland, OH) to remove residual primers and dNTPs following the manufacturer's protocol. The purified PCR products were subjected to Sanger sequencing using the APOE-Seq-F as the sequencing primer and an ABI PRISM^®^ BigDye Terminator Cycle Sequencing Ready Reaction Kit Version 3.1 (Perkin Elmer Applied Biosystems, Foster City, CA), according to the manufacturer's protocol. The sequences of the PCR primers and the size of PCR product for sequencing are listed in Table 1.

### Validation of the melting curve-based allele-specific PCR for APOE genotyping

To verify the accuracy of the melting curve-based allele-specific PCR for APOE genotyping, 300 DNA samples selected at random from 711 patients with schizophrenia and 665 control subjects were genotyped using the melting curve-based allele-specific PCR method and the Sanger sequencing method simultaneously. The genotyping results were compared between the two methods.

### Genetic association analysis of APOE with schizophrenia

After validation of the melting curve-based allele-specific PCR for APOE genotyping, we applied this method to conduct a genetic association study of APOE with schizophrenia consisting of 711 patients with schizophrenia and 665 control subjects. Differences in the genotype and allele frequency between the two groups were assessed using a chi-squared test. A P value less than 0.05 was considered statistically significant.

### APOE genotyping using Guthrie blood spot

To test the usefulness of the melting curve-based allele-specific PCR for APOE genotyping with DNA from different preparations, we first tested on whole blood samples collected on Guthrie filter paper (#903, Schleicher & Schuell, Inc., Keene, NH) for the screening of congenital errors of metabolism. A 3 mm diameter disc was punched from each Guthrie card and collected in the PCR tube. Each disc was immersed in a 50 microliter mixture of methanol and acetone (1:1 V/V) and dried at 60°C for 30 min. After drying, the disc was incubated in 50 microliter water at room temperature for at least 2 hours. Aliquots (4 microliter) of the solution were used for the melting curve-based allele-specific PCR of APOE genotyping method and the Sanger sequencing-based APOE genotyping method as described.

### APOE genotyping using whole blood without DNA purification

We further tested whether the whole blood lysate without DNA purification can be directly used in the melting curve-based allele-specific PCR method. Venous blood samples were collected using ethylenediaminetetraacetic acid (EDTA) as an anticoagulant, and frozen at -20°C freezer until use. After thawing and vortexing, the mixtures were centrifuged briefly. Aliquots (2 microliter) of crude blood lysate were first used directly as DNA template for the melting curve-based allele-specific PCR of APOE genotyping analysis as described before. Then, aliquots (2 microliter) of crude blood cell lysate were subjected to PCR amplification to generate an amplicon of 307 bp using primers APOE-Seq-F and APOE-158-R as described before. The 307 bp amplicons were subsequently subjected to APOE genotyping using the melting curve-based allele-specific PCR method and Sanger sequencing method as described.

## Results

### APOE genotyping using purified genomic DNA

**Fig 1:**
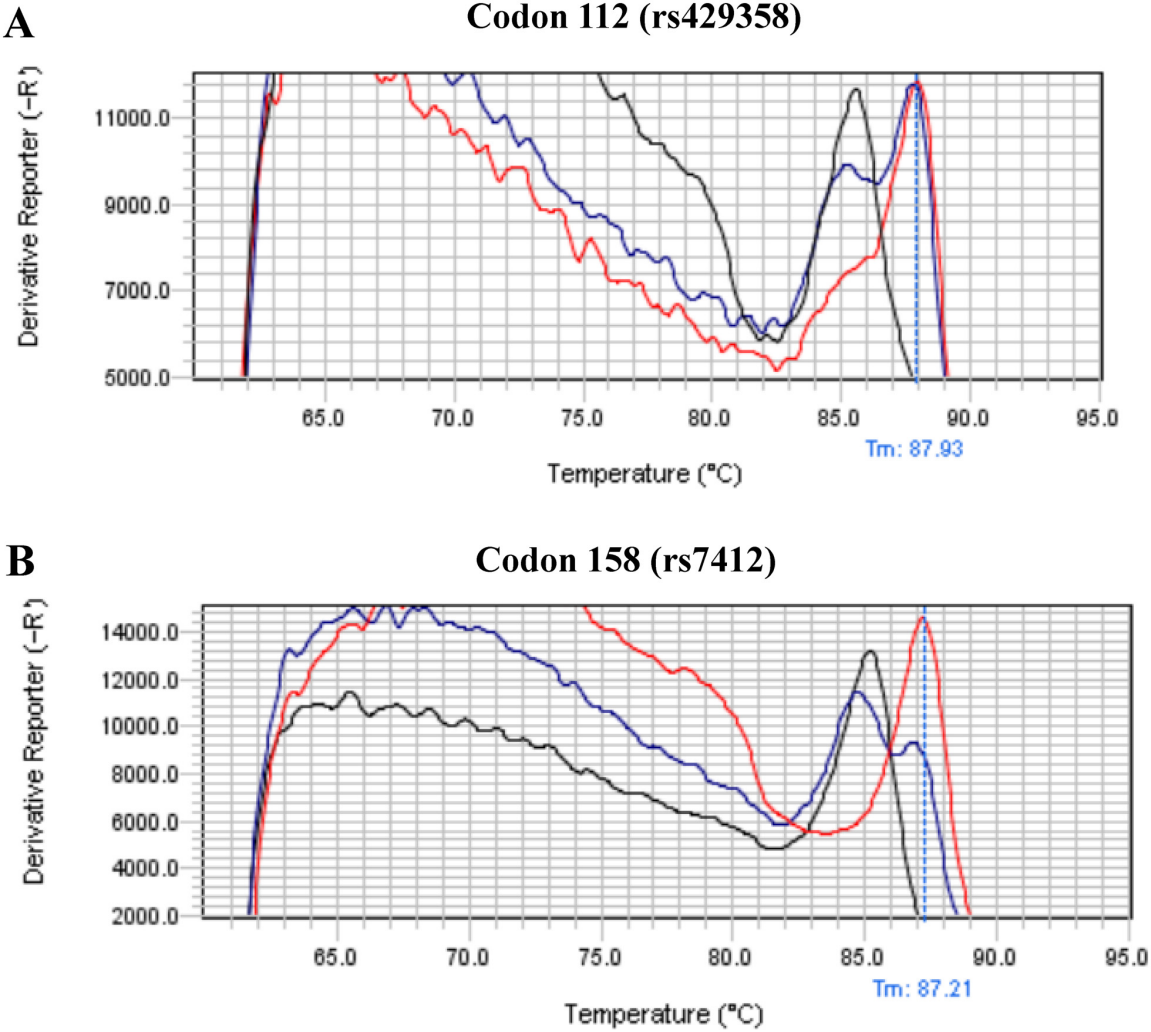
(A) Representative melting curve-based genotyping of rs429358 at codon 112 of APOE. Black color indicates TT homozygote; red color indicates CC homozygote while blue color indicates TC heterozygote. (B) Representative melting curve-based genotyping of rs7412 at codon 158 of APOE. Black color indicates TT homozygote; red color indicates CC homozygote while blue color indicates TC heterozygote.

The representative results of the melting curve-based allele-specific PCR genotyping of the codon 112 (rs429358) using purified genomic DNA are shown in Fig 1A. The TT homozygote had a distinct melting temperature from the CC homozygote while the TC heterozygote had two melting temperatures corresponding to that of TT and CC homozygote, respectively. Fig 1B demonstrates the representative results of the melting curve-based genotyping of the codon 158 (rs7412). The TT homozygote had a distinct melting temperature from the CC homozygote while the TC heterozygote had two melting temperatures corresponding to that of TT and CC homozygote, respectively.

### Validation of the melting curve-based allele-specific PCR for APOE genotyping

**Fig 2:**
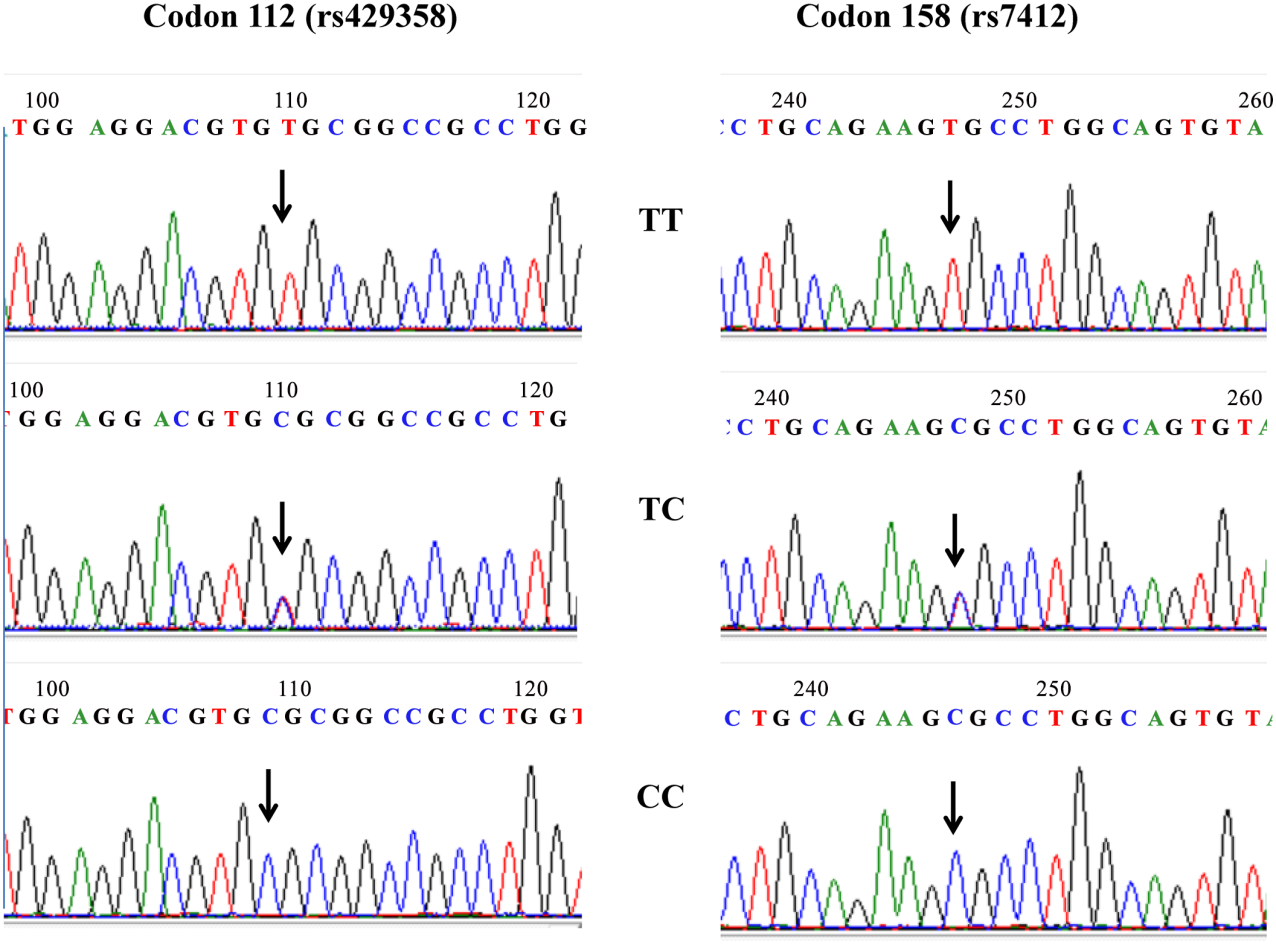
Traces of Sanger sequencing of APOE genotyping of codon 112 (rs429358) and codon 158 (rs7412). TT indicates homozygote of TT; TC indicates TC heterozygote, and CC indicates homozygote of CC. The arrow indicates the position of the single nucleotide polymorphism.

**Fig 3:**
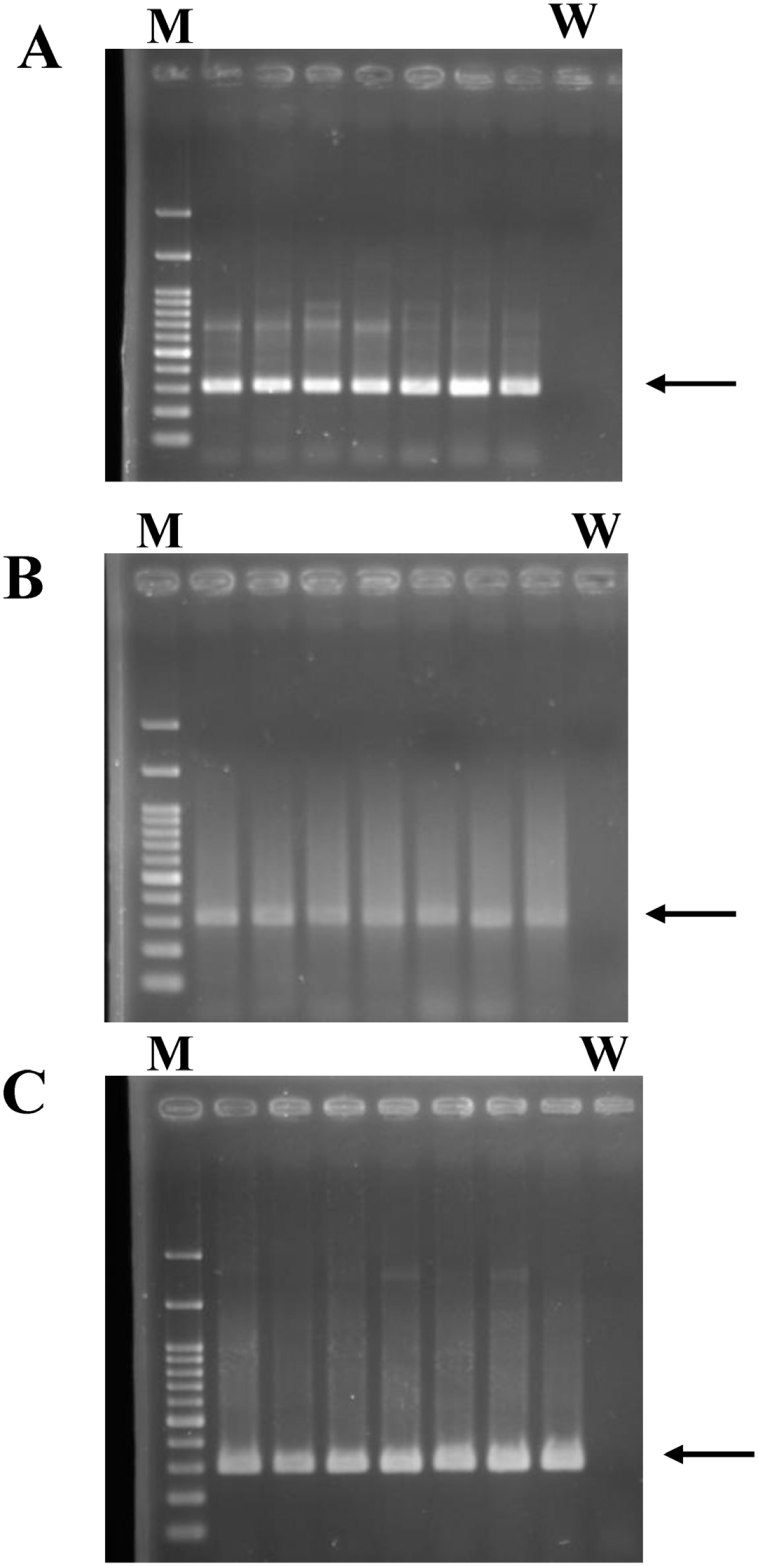
Representative gel electrophoresis of PCR amplicon using sequencing primers from different DNA preparations. (A) PCR amplicon generated from genomic DNA purified from peripheral blood cells. (B) PCR amplicon generated from Guthrie blood spot. (C) PCR amplicon generated from a crude lysate of peripheral blood cells (C). M: DNA markers; the arrow indicates the PCR amplicon with the size of 307 bp. W: indicates water blank as a negative control.

After genotyping a total of 300 DNA samples using both the melting curve-based allele-specific PCR method and the Sanger sequencing genotyping method simultaneously. We obtained a 100% concordance rate between these two methods. Representative sequencing results of are shown in Fig 2. Also, Fig 3A shows the PCR products generated from purified genomic DNA for Sanger sequencing using the sequencing primers.

### Genetic association analysis of schizophrenia

The melting curve-based allele-specific PCR method was applied to a genetic association study of APOE and schizophrenia in a sample of 711 patients with schizophrenia and 665 control subjects from Taiwan. The genotype and allele frequencies of these subjects are listed in Table 2. No significant differences in the genotype and allele frequencies were detected between these two groups.

**Table 2:** Genotype and allele frequencies of APOE2, E3 and E4 in this study.

	E2/E2	E2/E3	E2/E4	E3/E3	E3/E4	E4/E4	Total	p	E2	E3	E4	Total	p
Control								0.44					0.13
Counts	5	81	10	445	117	7	665		101	1088	141	1330	
%	0.8	12.2	1.5	66.9	17.6	1.1	100		7.6	81.8	10.6%	100	
Schizophrenia													
Counts	3	84	10	507	103	4	711		100	1201	121	1422	
%	0.4	11.8	1.4	71.3	14.5	0.6	100		7.0	84.5	8.5	100	

### APOE genotyping using Guthrie blood spot and whole blood

A total of 10 Guthrie cards containing blood spots collected from 10 individuals were tested in this study. The storage duration of these cards ranged from 18–20 years. Aliquots of solution that contained the dissolved genomic DNA from Guthrie blood spot were directly subjected to APOE genotyping using the melting curve-based allele-specific PCR method. We were able to determine the APOE genotype of these ten individuals using this method (data not shown). The authenticity of the genotype of these ten individuals was verified using Sanger sequencing method that used aliquots of the same DNA solution collected from Guthrie card. Fig 3B shows the PCR amplicon of 307 bp using the sequencing primers APOE-Seq-F and APOE-158-R.

In the experiment of using crude blood cell lysate without purification as DNA template for APOE genotyping. A total of 10 samples collected from 10 individuals were tested in this study. At first, we were not able to directly determine the APOE genotyping using the melting curve-based allele-specific PCR method. Then, we PCR amplified the crude blood cell lysate using the primer pairs APOE-Seq-F and APOE-158-R and PCR conditions described in method section. As shown in Fig 3C, the 307 bp PCR products were successfully obtained from aliquots (2 microliter) of crude blood cell lysate. Aliquots of the amplicons were further subjected to APOE genotyping using the melting curve-based allele-specific PCR method and the Sanger sequencing method successfully (data not shown).

## Discussion

In this study, we developed an APOE genotyping method based on the principle of Tm-shift SNP genotyping method described by Wang and colleagues [30]. In this method, two allele-specific forward PCR primers were tagged with two different lengths of GC tail, respectively, which generated two allele-specific PCR amplicons with different sizes. As melting temperature of PCR amplicon is size dependent, the two different sizes of allele-specific amplicons result in two distinct melting temperatures in dissociation curve analysis. Hence, this method has a high resolution in the determination of the SNP genotype of APOE. Our method used dissociation curve analysis of default setting of the machine and did not need to use dedicated software. Hence, the protocol should be easily adopted in other machines. The success of our protocol also lends further support to the general utility of the Tm-shift SNP genotyping method in selective SNPs of interest [30]. Nevertheless, as shown in Fig 1, there is a high baseline before the peak of melt temperature, which might be due to the presence of some non-specific PCR products. Although the high baseline does not affect the accuracy of the APOE genotype, the current melting curve-based allele-specific PCR method for APOE genotyping needs further optimization certainly.

In the validation study, a 100% concordance rate was obtained by the melting genotyping method and the Sanger sequencing method in a sample of 300 subjects, indicating that the melting curve-based allele-specific PCR assay is as accurate as Sanger sequencing method. But our method is faster and more cost savings than the Sanger sequencing method. Furthermore, when our method was applied to the genetic association study of APOE polymorphisms and schizophrenia in our population, the genotyping of 711 patients with schizophrenia and 665 healthy control subjects was completed in one month by one research assistant, suggesting this is an efficient APOE genotyping method.

Nevertheless, no association of APOE genotypes with schizophrenia was observed in this study. Several case-control studies have been conducted on the genetic association of APOE and schizophrenia. The results have been inconsistent and equivocal in different studies [12, 31]. In a meta-analysis of the genetic association of APOE with schizophrenia that consisted of 11 Caucasian and 6 Asian case-control studies, APOE4 was found to have a modest association of risk with schizophrenia in the Caucasian population, but not in Asian population [12]. Furthermore, no other APOE alleles were found to be associated with schizophrenia in this meta-analysis in either Caucasian or Asian samples when analyzed separately or together [12]. In a recent systematic review and updated meta-analysis of the genetic association of APOE with schizophrenia that consisted of 28 studies, the authors reported a significant protective effect of APOE3 in the Asian population (OR = 0.73, 95% CI = 0.54–0.98) [31]. The other alleles were not found to have a significant association in their study [31]. We did not observe the protective effective of APOE3 against schizophrenia in our present study, nor did we observe the association of other alleles of APOE with schizophrenia. Nevertheless, the genetic data of our study shall contribute to future meta-analysis study of APOE and schizophrenia. Also, our genetic data would be useful for the case-control association study of APOE genotype with other diseases such as type 2 diabetes and coronary artery disease in our population [32].

In this study, we also demonstrated that blood samples collected on Guthrie blood filter paper that were used in the screening program of congenital errors of metabolism can be used directly in the melting curve-based allele-specific PCR APOE genotyping assay. Based on this result, we suggest that APOE genotyping can be added to the items of newborn screening programs to detect infants with a high risk of Alzheimer’s disease, cardiovascular diseases, and type 2 diabetes. Thus, preventive procedures may be adopted to avoid or delay the onset of these diseases in their later life.

In the attempt of using total cell lysate of peripheral blood cells without DNA purification for APOE genotyping, we failed to use the crude lysate as DNA template directly for the melting curve-based allele-specific PCR APOE genotyping. The causes might be due to some materials present in the crude blood cell lysate that may inhibit PCR and interfere with the fluorescence detection. Further troubleshooting is needed to overcome this issue. Despite this, we were able to obtain PCR amplicon from the crude blood cell lysate using the sequencing primers APOE-Seq-F and APOE-158-R. The amplicon was used successfully for downstream melting curve-based allele-specific PCR and Sanger sequencing-based APOE genotyping. Thus, although indirectly, the use of crude blood cell lysate in the melting curve-based allele-specific PCR APOE genotyping represents a fast and cost-saving method that does not need DNA purification from blood cells. Hence, this is a convenient method and cost saving method that can be used in a large-scale population study.

In summary, we developed a melting curve-based allele-specific PCR method for APOE genotyping that is efficient, convenient, and accurate and has the flexibility to suit different sample size. The method is versatile and robust that different preparations of DNA can be used in this method. Thus, the method should potentially have a broad range of applications in clinical and research laboratories.

## Abbreviations

APOE: apolipoprotein E
bp: base pair
EDTA: ethylenediaminetetraaceitc acid
PCR: FRET: fluorescent resonance energy transfer
HRM: high-resolution melting
PCR: polymerase chain reaction
RFLP: restriction fragment length polymorphism
SNP: single nucleotide polymorphism
Tm: melting temperature

## References

[1] Ozder A. Lipid profile abnormalities seen in T2DM patients in primary healthcare in Turkey: a cross-sectional study. Lipids Health Dis. 2014;13:183 10.1186/1476-511X-13-183 25481115 PMC4271485

[2] Phillips MC. Apolipoprotein E isoforms and lipoprotein metabolism. IUBMB Life. 2014;66(9):616–23. 10.1002/iub.1314 . 25328986

[3] Villeneuve S, Brisson D, Marchant NL, Gaudet D. The potential applications of Apolipoprotein E in personalized medicine. Frontiers in aging neuroscience. 2014;6:154 10.3389/fnagi.2014.00154 25071563 PMC4085650

[4] Lopez MF, Krastins B, Ning M. The role of apolipoprotein E in neurodegeneration and cardiovascular disease. Expert Rev Proteomics. 2014;11(3):371–81. 10.1586/14789450.2014.901892 . 24754513

[5] Ward A, Crean S, Mercaldi CJ, Collins JM, Boyd D, Cook MN, et al Prevalence of apolipoprotein E4 genotype and homozygotes (APOE e4/4) among patients diagnosed with Alzheimer's disease: a systematic review and meta-analysis. Neuroepidemiology. 2012;38(1):1–17. 10.1159/000334607 . 22179327

[6] Liu Y, Yu JT, Wang HF, Han PR, Tan CC, Wang C, et al APOE genotype and neuroimaging markers of Alzheimer's disease: systematic review and meta-analysis. J Neurol Neurosurg Psychiatry. 2015;86(2):127–34. 10.1136/jnnp-2014-307719 24838911 PMC4331076

[7] Yu JT, Tan L, Hardy J. Apolipoprotein E in Alzheimer's disease: an update. Annu Rev Neurosci. 2014;37:79–100. 10.1146/annurev-neuro-071013-014300 . 24821312

[8] Sutcliffe JG, Thomas EA. The neurobiology of apolipoproteins in psychiatric disorders. Mol Neurobiol. 2002;26(2–3):369–88. . 12428765 10.1385/mn:26:2-3:369

[9] Gibbons AS, Udawela M, Jeon WJ, Seo MS, Brooks L, Dean B. The neurobiology of APOE in schizophrenia and mood disorders. Front Biosci (Landmark Ed). 2011;16:962–79. . 21196212 10.2741/3729

[10] Feng F, Lu SS, Hu CY, Gong FF, Qian ZZ, Yang HY, et al Association between apolipoprotein E gene polymorphism and depression. J Clin Neurosci. 2015;22(8):1232–8. 10.1016/j.jocn.2015.02.012 . 25979253

[11] Skoog I, Waern M, Duberstein P, Blennow K, Zetterberg H, Borjesson-Hanson A, et al A 9-year prospective population-based study on the association between the APOE*E4 allele and late-life depression in Sweden. Biol Psychiatry. 2015;78(10):730–6. 10.1016/j.biopsych.2015.01.006 . 25708227

[12] Xu MQ, St Clair D, He L. Meta-analysis of association between ApoE epsilon4 allele and schizophrenia. Schizophr Res. 2006;84(2–3):228–35. 10.1016/j.schres.2006.02.015 . 16567081

[13] Liu W, Breen G, Zhang J, Li S, Gu N, Feng G, et al Association of APOE gene with schizophrenia in Chinese: a possible risk factor in times of malnutrition. Schizophr Res. 2003;62(3):225–30. . 12837518 10.1016/s0920-9964(02)00384-5

[14] Allen NC, Bagade S, McQueen MB, Ioannidis JP, Kavvoura FK, Khoury MJ, et al Systematic meta-analyses and field synopsis of genetic association studies in schizophrenia: the SzGene database. Nat Genet. 2008;40(7):827–34. 10.1038/ng.171 . 18583979

[15] Soeira-de-Souza MG, Bio DS, Dias VV, Martins do Prado C, Campos RN, Costa LF, et al SHORT COMMUNICATION: Apolipoprotein E genotype and cognition in bipolar disorder. CNS Neurosci Ther. 2010;16(5):316–21. 10.1111/j.1755-5949.2010.00153.x . 20406267 PMC6493783

[16] Kessing LV, Jorgensen OS. Apolipoprotein E-epsilon 4 frequency in affective disorder. Biol Psychiatry. 1999;45(4):430–4. . 10071713 10.1016/s0006-3223(98)00038-9

[17] Bellivier F, Laplanche JL, Schurhoff F, Feingold J, Feline A, Jouvent R, et al Apolipoprotein E gene polymorphism in early and late onset bipolar patients. Neurosci Lett. 1997;233(1):45–8. . 9324236 10.1016/s0304-3940(97)00624-1

[18] Giunco CT, de Oliveira AB, Carvalho-Salles AB, Souza DS, Silva AE, da Rocha SS, et al Association between APOE polymorphisms and predisposition for autism. Psychiatr Genet. 2009;19(6):338 . 19859026 10.1097/YPG.0b013e3283328e41

[19] Ashley-Koch AE, Jaworski J, Ma de Q, Mei H, Ritchie MD, Skaar DA, et al Investigation of potential gene-gene interactions between APOE and RELN contributing to autism risk. Psychiatr Genet. 2007;17(4):221–6. . 17621165 10.1097/YPG.0b013e32809c2f75

[20] Persico AM, D'Agruma L, Zelante L, Militerni R, Bravaccio C, Schneider C, et al Enhanced APOE2 transmission rates in families with autistic probands. Psychiatr Genet. 2004;14(2):73–82. . 15167692 10.1097/01.ypg.0000128768.37838.17

[21] Gatt JM, Burton KL, Williams LM, Schofield PR. Specific and common genes implicated across major mental disorders: a review of meta-analysis studies. J Psychiatr Res. 2015;60:1–13. 10.1016/j.jpsychires.2014.09.014 . 25287955

[22] Martinez-Barrondo S, Saiz PA, Morales B, Garcia-Portilla MP, Coto E, Alvarez V, et al Negative evidences in association between apolipoprotein E polymorphism and panic disorder. Eur Psychiatry. 2006;21(1):59–61. 10.1016/j.eurpsy.2005.04.012 . 15961292

[23] Johnson LA, Zuloaga DG, Bidiman E, Marzulla T, Weber S, Wahbeh H, et al ApoE2 Exaggerates PTSD-Related Behavioral, Cognitive, and Neuroendocrine Alterations. Neuropsychopharmacology. 2015;40(10):2443–53. 10.1038/npp.2015.95 25857685 PMC4538360

[24] Lyons MJ, Genderson M, Grant MD, Logue M, Zink T, McKenzie R, et al Gene-environment interaction of ApoE genotype and combat exposure on PTSD. Am J Med Genet B Neuropsychiatr Genet. 2013;162B(7):762–9. 10.1002/ajmg.b.32154 . 24132908 PMC4745646

[25] Kassam I, Gagnon F, Cusimano MD. Association of the APOE-epsilon4 allele with outcome of traumatic brain injury in children and youth: a meta-analysis and meta-regression. J Neurol Neurosurg Psychiatry. 2015 10.1136/jnnp-2015-310500 . 25904811

[26] Martorell L, Virgos C, Valero J, Coll G, Figuera L, Joven J, et al Schizophrenic women with the APOE epsilon 4 allele have a worse prognosis than those without it. Mol Psychiatry. 2001;6(3):307–10. 10.1038/sj.mp.4000855 . 11326299

[27] Rihn BH, Berrahmoune S, Jouma M, Chamaa S, Marcocci L, Le Faou A. APOE genotyping: comparison of three methods. Clin Exp Med. 2009;9(1):61–5. 10.1007/s10238-008-0012-2 . 18843526

[28] Zhan XH, Zha GC, Jiao JW, Yang LY, Zhan XF, Chen JT, et al Rapid identification of apolipoprotein E genotypes by high-resolution melting analysis in Chinese Han and African Fang populations. Exp Ther Med. 2015;9(2):469–75. 10.3892/etm.2014.2097 25574218 PMC4280925

[29] Calero O, Hortiguela R, Bullido MJ, Calero M. Apolipoprotein E genotyping method by real time PCR, a fast and cost-effective alternative to the TaqMan and FRET assays. J Neurosci Methods. 2009;183(2):238–40. 10.1016/j.jneumeth.2009.06.033 . 19583979

[30] Wang J, Chuang K, Ahluwalia M, Patel S, Umblas N, Mirel D, et al High-throughput SNP genotyping by single-tube PCR with Tm-shift primers. BioTechniques. 2005;39(6):885–93. . 16382908 10.2144/000112028

[31] Gonzalez-Castro TB, Tovilla-Zarate CA, Hernandez-Diaz Y, Fresan A, Juarez-Rojop IE, Ble-Castillo JL, et al No association between ApoE and schizophrenia: Evidence of systematic review and updated meta-analysis. Schizophr Res. 2015;169(1–3):355–68. 10.1016/j.schres.2015.08.031 . 26372448

[32] Chaudhary R, Likidlilid A, Peerapatdit T, Tresukosol D, Srisuma S, Ratanamaneechat S, et al Apolipoprotein E gene polymorphism: effects on plasma lipids and risk of type 2 diabetes and coronary artery disease. Cardiovasc Diabetol. 2012;11:36 10.1186/1475-2840-11-36 22520940 PMC3372424

